# Elbow hemiarthroplasty with a 3D-printed prosthesis for distal humeral bone defects after tumor excision: a case report

**DOI:** 10.1186/s41205-023-00178-8

**Published:** 2023-06-14

**Authors:** Yingkang Zhu, Shuo Gong, Jin Dai, Lei Zhou

**Affiliations:** Department of orthopedic and soft tissue surgery, Shandong Cancer Hospital and Institute, Shandong First Medical University, Shandong Academy of Medical Sciences, Jinan, 250117 Shandong Province China

**Keywords:** Distal humerus, 3D-printed prosthesis, Hemiarthroplasty

## Abstract

**Introduction:**

The distal humerus is a rare site for primary and metastatic bone tumors. Due to the scarcity of cases and lack of standardized surgical strategies, it is often difficult for surgeons to choose the right choice. The application of a 3D-printed prosthesis with hemiarthroplasty for the treatment of the distal humerus after tumor resection can be a very effective option.

**Case presentation:**

We present a clinical case of a 3D-printed distal humeral prosthesis for the treatment of bone defects caused by metastatic bone tumors. The preoperative evaluation was aggressively performed, and the decision was made to distal humeral hemiarthroplasty (DHH) after wide resection of the tumor segment bone. Processing of the Digital Imaging and Communications in Medicine (DICOM) data from CT scans performed after mirror conversion using CT data of the contralateral humerus, we designed a 3D-printed distal humeral prosthesis with hemiarthroplasty. After reconstruction of bone and surrounding soft tissue by the 3D-printed prosthesis combined with the LARS ligament and regular follow-up for 12 months, the patient had an MSTS-93 score of 29 and an MEP of 100, which reached a good level, and the patient was fully competent in normal daily activities.

**Conclusions:**

Our results show that the 3D-printed modular prosthesis with hemiarthroplasty is a very effective option for cases of large elbow bone defects due to primary bone tumors or metastatic disease. However, careful preoperative preparation is required for the best outcome. Careful preoperative preparation and long-term follow-up are essential for the best outcome.

## Introduction

The distal humerus is an uncommon rare site for primary bone tumors or metastatic disease. Investigators conducting retrospective reviews reported that only 91 (1.2%) of 7830 primary malignant bone lesions occurred in the distal humerus and elbow region [1]. Although distal humeral neoplastic lesions are rare, limb salvage treatment of distal humeral tumors remains a clinical challenge for orthopedic surgeons [2, 3]. The position of the elbow is superficial and is inherently unstable anatomy, requiring complex joint structures including the humeroulnar joint、humeroradial joint、proximal radioulnar joint, and dynamic stabilization systems including the brachial and forearm muscles to maintain performance. In addition, the treatment of malignant bone tumors is more challenging than in other anatomic regions due to the limited soft tissue envelope and neurovascular structures close to the tumor in the elbow region [2]. These complex tumor invasion conditions could lead to persistent pain, residual instability, or functional disability from stiffness. The main goal of surgery for malignant tumors of the elbow is resection with clear margins, but it similarly creates an intractable bone defect problem [4]. There is no consensus on the optimal reconstructive technique following distal humeral resection. Patient mobility, tumor characteristics, and the degree of anatomic involvement are important factors to consider when choosing the best reconstructive modality [5]. Autograft has previously been considered an effective strategy for treating bone defects, but it has limited clinical application because of the need for additional surgery to obtain bone intraoperatively and the potential for additional complications such as pain, infection, fracture, and nerve injury at the site of removal [6]. Similarly, allograft reconstruction has many risks of inducing an immune response, integrating with host bone, remodeling slowly, and transmitting diseases [7]. In large segmental bone defects, elbow arthrodesis is difficult to achieve and patient acceptance and functional outcomes tend to be poor [8]. As the prognosis of the allograft-prosthetic composite (APC) and prosthesis reconstructive procedure continues to improve, total elbow arthroplasty (TEA) is becoming more common, with the advantages of immediate stabilization, early mobility, and satisfactory functional status, and has now become the primary strategy for reconstruction [1, 9]. However, elbow prostheses for oncological situations are mostly hinged articulation, which will cause greater stress at the liner and around the intramedullary stem during flexion-extension activities of the elbow, and eventually causing wear and loosening of the prosthesis, producing mechanical complications resulting from altered stress [3, 10]. To reduce prosthetic stress and complications, distal humeral hemi-arthroplasty (DHH) based on three-dimensional (3D) printing technology may be a reasonable approach. In 3D visualization, human tissue anatomy is displayed on the screen, and 3D printing extends this representation to physical objects that are conducive to patient diagnosis and treatment [11]. 3D printing technology can produce the same metallic humerus as the original bony articular surface, achieving biomimetic reconstruction purposes and completely mimicking elbow static versus dynamic physiological states.

We share a patient with a metastatic tumor of the distal humerus with pathological fracture after successfully utilizing DHH treatment, who underwent reconstruction with a construct designed for joint stabilization and augmentation with a 3D-printed distal humeral prosthesis (Chunli, Beijing China). The objective is to investigate the perioperative safety evaluation, prosthesis-related complications, and postoperative functional status of DHH using a 3D-printed prosthesis. We obtained written informed consent from all patients. This case series has been reported by SCARE Guidelines [12].

## Case presentation

**Fig. 1:**
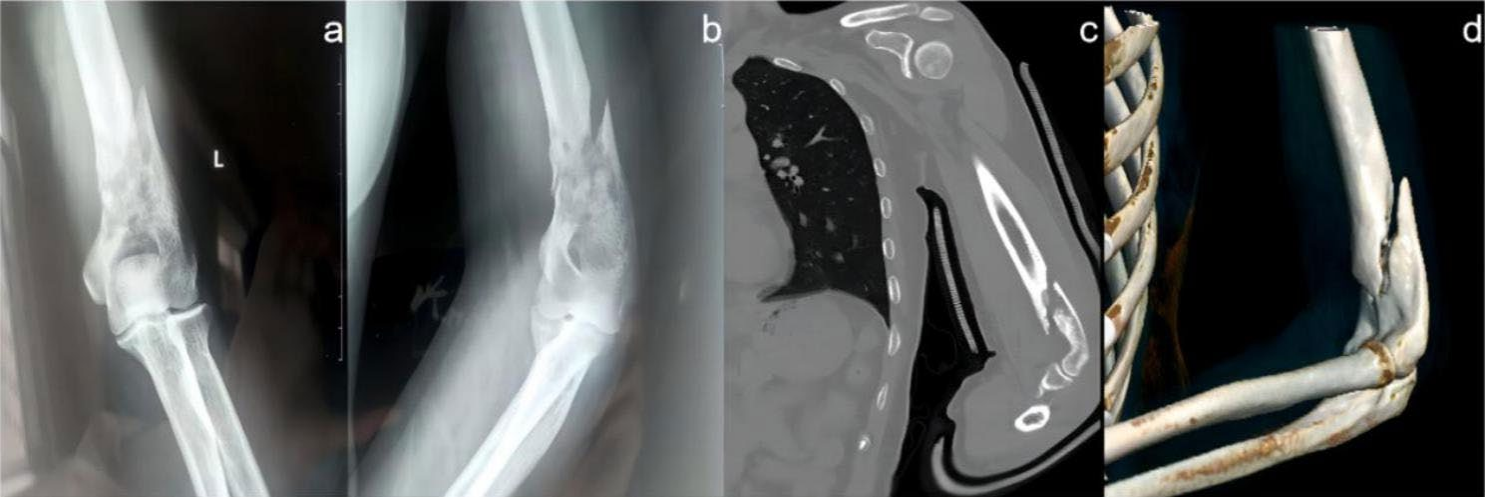
The AP view (**a**) and lateral view (**b**) X-rays before surgery showed bone destruction at the lower end of the left humerus, discontinuous bone cortex, and slightly angulated. More clearly CT coronal scans (**c**) and 3D CT reconstructions (**d**) demonstrated the fracture line and suggested a visible soft tissue density shadow at the lower humerus with surrounding as well as subcutaneous soft tissue swelling suggestive of a pathologic fracture

A 49-year-old female patient who has been diagnosed with breast cancer for over 8 years presented with left upper extremity pain and swelling with limited activity for 20 days after lifting heavy loads. The circumstances for the patient to come to this visit were as follows: no obvious swelling and deformity of the left upper extremity, marked atrophy of all muscles, marked tenderness over the elbow joint, palpable bone rub sensation, positive pain on longitudinal percussion, position of the posterior triangle of the elbow and active and passive activity of the left elbow could not be examined due to pain, distal muscle strength of the left upper extremity was reduced due to pain, left-hand activity, sensation, revascularization, and no significant abnormalities. Preoperative radiographs and 3D-CT scans showed broken bone lines, suggestive of pathologic fracture (Fig. 1).

**Fig. 2:**
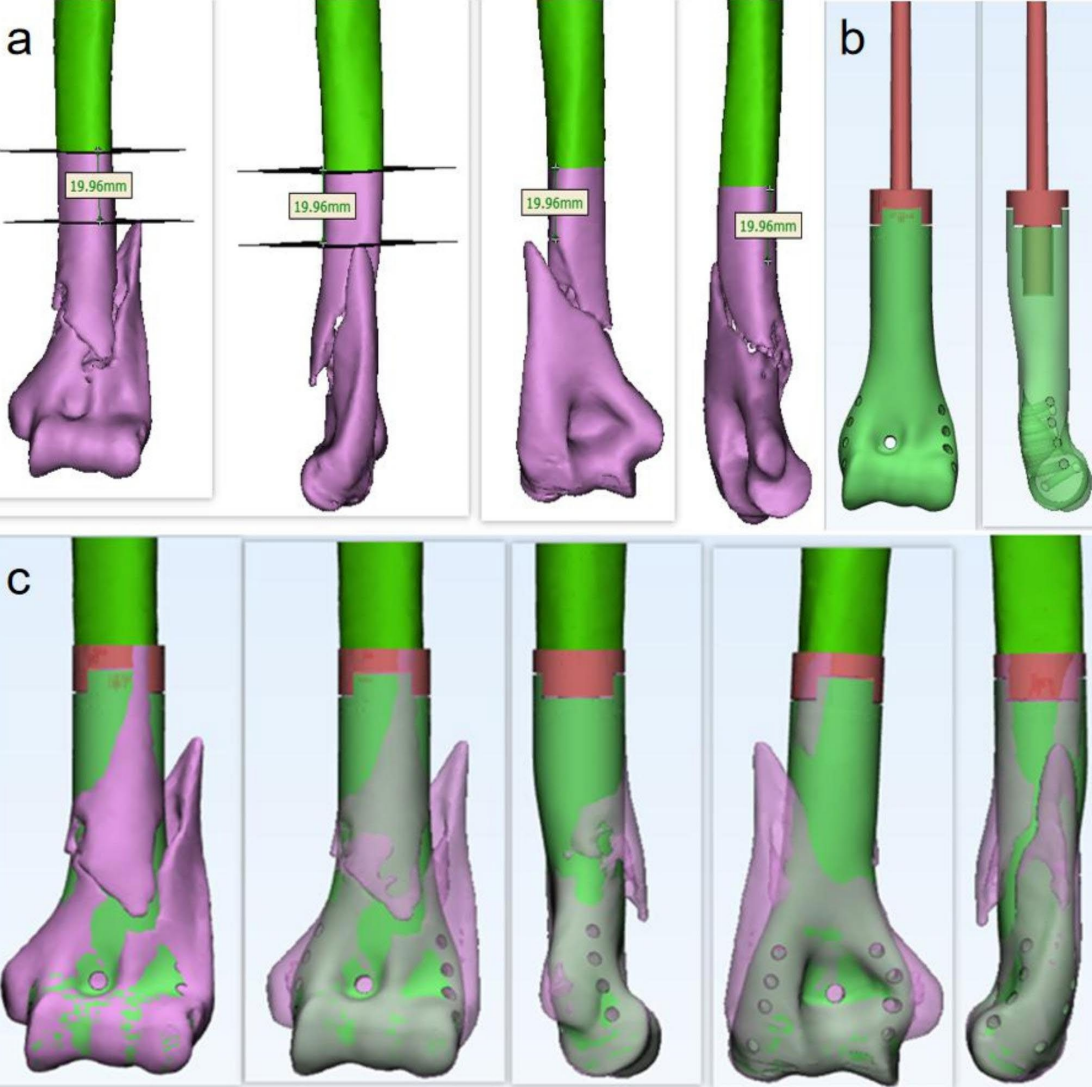
Design of the 3D-printed prostheses with hemiarthroplasty for the distal humerus. Based on the radiographic appearance of the patient, preoperative discussion determined a safe osteotomy length of 20 mm proximal to the fracture line (**a**). The proximal end is a cement-type medullary pin and the distal prosthesis is a 3D print with four side holes at each edge of the prosthesis and one central hole (**b**). The profile of the prosthesis of the mirror design was compared with the portion of the patient’s distal humerus that required resection (**c**)

We decided to perform a wide excision of the area of the intrasegmental bone adducted fracture of the distal humerus and perform a DHH to reconstruct the distal humeral bone defect with a 3D-printed distal humeral prosthesis. She underwent bilateral humeral CT thin-section scans as well as enhanced MRI imaging of the humerus on the affected side preoperatively. Based on patient radiographic findings, preoperative discussion determined a safe osteotomy length of 20 mm proximal to the talar crease (Fig. 2a). The Digital Imaging and Communications in Medicine (DICOM) data from CT scans performed to facilitate personalized prosthesis design after the CT data of the contralateral humerus were mirror transformed (Fig. 2b).

**Fig. 3:**
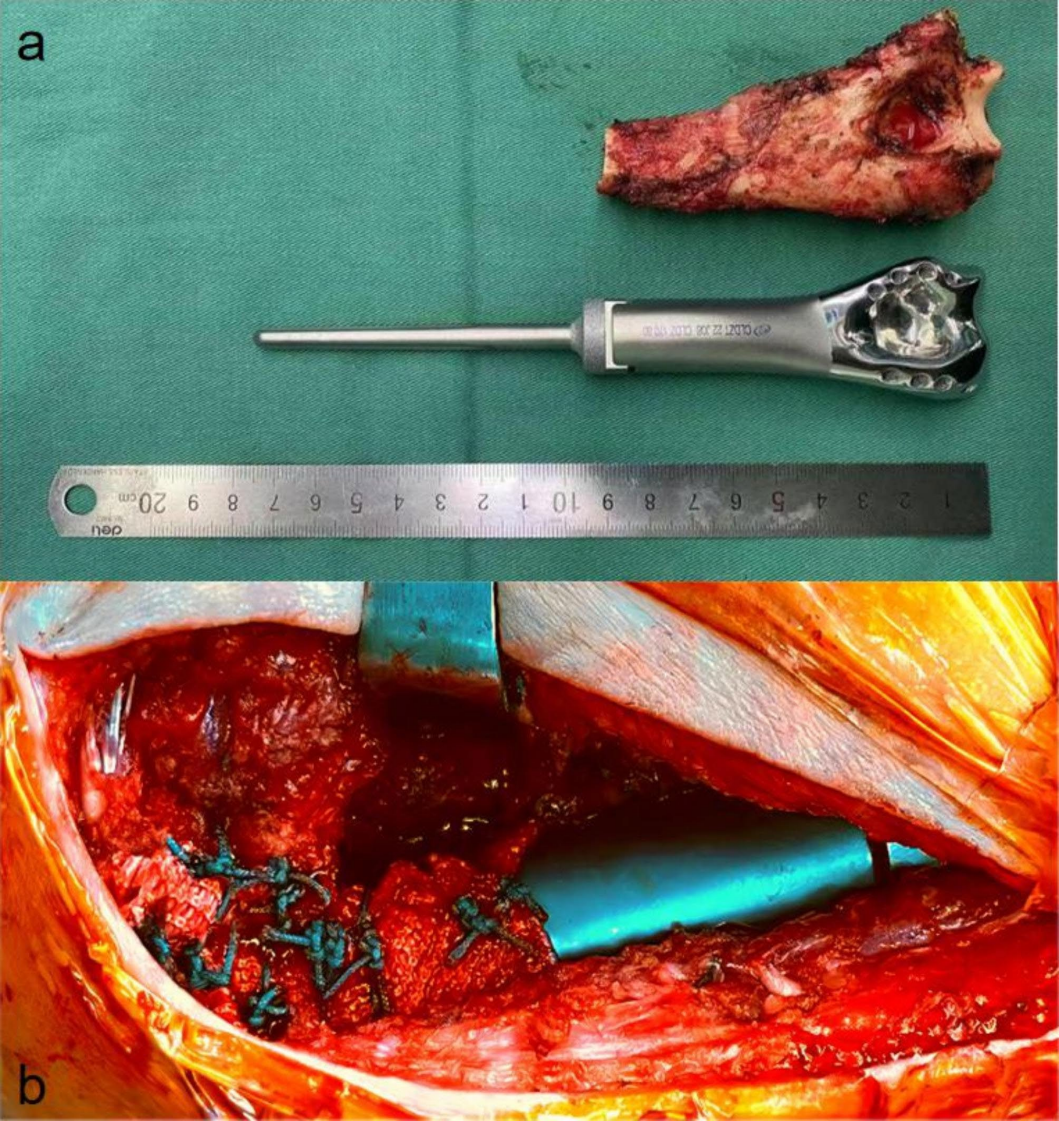
Intraoperative photographs of gross tumor resection specimens (**a**) and prosthetic implantation (**b**)

After the patient received general anesthesia, we marked the body surface incision position and exposed the ulnar nerve after careful dissection of soft tissue. From the distal humeral surface to the triceps tendon attachment, the triceps tendon was semi-dissected to preserve the attachment point, the elbow joint was exposed, and the position of the distal humerus fracture line was fully exposed with a macroscopically negative resection margin of 2 cm above the fracture line, at which the humerus was detached according to the preoperative plan. We performed distal humeral reshaping and processed the medullary canal of the humerus. After the prosthesis was assembled in vitro, it was wrapped with an artificial ligament (LARS; Laboratoired’Application et de Recherche Scientifique, France). After the prosthesis is fixed to the humerus by cementation, the joint capsule was carefully sutured to the LARS ligament, and the anterior joint capsule was sutured to the prosthetic central hole. The collateral ligaments and common tendons of the flexors and extensors were sutured to the medial and lateral condylar nail holes, respectively (Fig. 3). Postoperative pathology was that the tumor segment bone of the distal left humerus was consistent with breast cancer metastasis with negative resection margins.

**Fig. 4:**
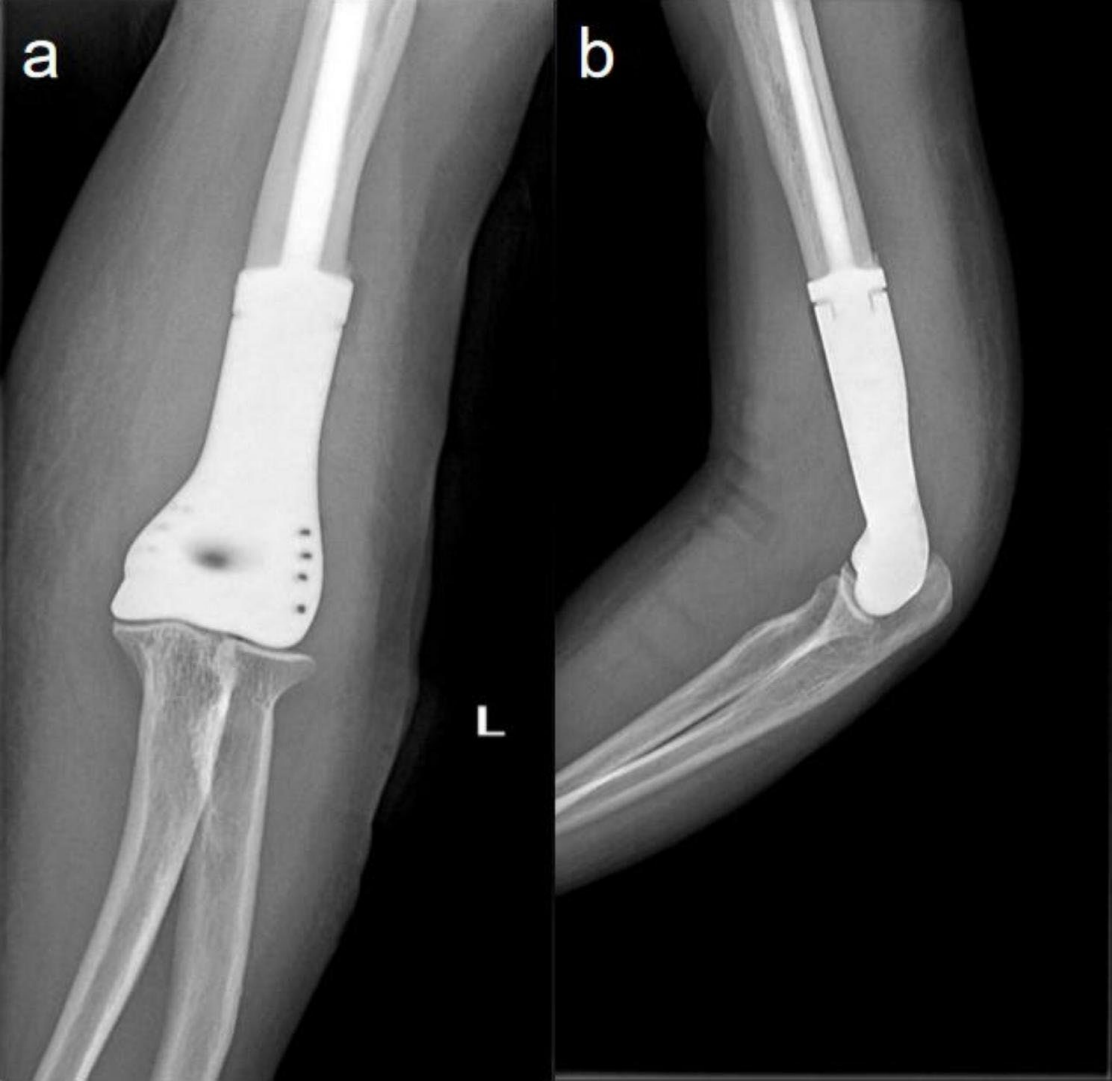
Follow-up elbow AP view (**a**) and lateral view (**b**) X-rays after surgery showed excellent alignment of the articular surfaces and good positioning of the prosthesis, with no evidence of loosening, dislocation, or arthritis

Patients were allowed to exercise their shoulders and hands immediately postoperatively. Passive flexion-extension movements of the elbow were prescribed within a week postoperatively. Two weeks postoperatively, active movements were initiated, including flexion, extension, pronation, and supination activities. The patient was scheduled for regular follow-up every 3 months. 12 months after surgery: complete healing of surgical scar with active flexion 130 °and active extension 0 °of elbow. A good level was achieved with the Musculoskeletal Tumor Society- 93 (MSTS-93) score of 29 and the Mayo Elbow Performance Score (MEPs) of 100, and the patient was able to resume normal daily activities. Follow-up elbow AP view (a) and lateral view (b) X-rays after surgery showed satisfactory results (Fig. 4).

## Discussion

The indications for performing a distal humeral replacement are broad, ranging from bone and soft tissue sarcomas, bone metastases, multiple myeloma, and benign bone tumors to patients with posttraumatic bone defects or failed previous joint replacement [13, 14]. Megaprosthesis and allograft-prosthetic composite (APC) have been the mainstay of reconstruction for the treatment of distal humeral and proximal ulnar defects, but the concept of hemiarthroplasty has rarely been adopted and its use in oncologic cases has been reported in only small case series [5, 15]. Elbow surgery is associated with a greater likelihood of developing major complications after trauma, such as severe elbow degeneration, complete elbow joint stiffness, elbow adhesion, distal humeral delayed union or nonunion and bone defects [14]. Therefore for elbow surgery, many authors believe that elbow arthroplasty is the best solution to restore elbow anatomy and function [14, 16]. We suggested that mirror 3D printed DHH is the best method to help restore elbow anatomy and range of motion when patients have pathological fractures or even large segmental bone defects with very poor surrounding soft tissue or poor bone quality that is difficult to work with other methods. Because bone stock is reduced under the conditions described above, traditional elbow prostheses are insufficient to restore a functional state of the joint [9]. Our patient underwent distal humeral prosthesis with hemiarthroplasty based on a personalized 3D-printed prosthesis design protocol, and after a 12-month follow-up, this approach achieved a good perioperative safety profile, a low incidence of prosthetic complications, and satisfactory functional outcomes.

3D printing has played an increasingly important role in achieving precision medicine. Customized implants can generate 3D data, allowing for pre-virtual surgeries to be carried out and designed to mimic the contralateral healthy side of a customized implant [17]. This expands the planning and navigation of orthopedic surgeries and contributes to new surgical methods for osteotomy, fracture fixation, and joint replacement [18]. In addition, preoperative simulation surgery can be performed on physical 3D models, allowing for more intuitive problem-solving and measurement. 3D printing improves preoperative planning, provides a superior mechanism to visualize and examine potential pathological conditions, and can effectively educate patients and healthcare teams. This plan has changed the surgical management of some patients and become a distinct alternative method. Preoperative planning can also reduce operating room time and the number of equipment and tools that need to be attempted, subsequently wasted, and/or re-disinfected [18]. In this sense, 3D printing has been proven to be quite beneficial for diagnosing orthopedic pathology and surgical intervention.

Currently, most implants used for reconstruction after resection of a periarticular tumor are linked prostheses with TEA [1, 9, 19]. Although the linked articulation design ensures joint stability and a good range of motion, it limits the direction of joint movement, leading to increased stress at the joint interface and the manubrium, producing common complications after elbow replacement, including aseptic loosening, socket wear, and periprosthetic fracture [20]. The patient’s joint mobility, tumor biology, and the degree of anatomic involvement are important factors to consider when choosing the best reconstructive modality. Therefore, to increase the life expectancy of patients as well as increase the quality of life, we should overcome the stress-generated disadvantages of TEA. 3D printing the congruence of a proximal DHH with the distal articular surface can reduce contralateral bone wear, which can reduce the risk of degenerative or traumatic osteoarthritis and traumatic myositis ossificans [21]. Therefore, the 3D-printed DHH not only achieves the short-term requirements of joint surface matching, joint stability, and mobility but also reduces the periprosthetic stress and solves the critical issues of wear and loosening in the long term.

Although DHH is a good option for large bone defects or severe elbow injury to help patients recover the anatomical shape and function of the elbow joint, postoperative complication rates have rarely been reported by investigators due to the disease and the scarcity of surgical modalities [1]. In addition, although the preliminary results showed a good functional recovery without mechanical complications, a longer follow-up is needed because usually, many complications do not occur in the first two years. However, the authors believe that because patients have severe bone and soft tissue damage preoperatively, they are at higher risk for postoperative complications such as artificial loosening, infection, and nerve injury. Our patient showed satisfactory recovery of joint function without the above complications, which may be attributed to the presence of a simple fracture at the distal humerus, intact articular surface of the ulna and radius, and milder tumor tissue invasion force. Therefore, the patient had a 3D-printed proximal hemielbow prosthesis that matched bone and soft tissue good conditions that favored surgical reconstruction of the hemi elbow, postoperative recovery, and reduced complications. Therefore, the patient had a 3D-printed prosthesis with hemiarthroplasty matched bone and soft tissue good conditions, which were more favorable for surgical reconstruction of the DHH, postoperative recovery as well as reducing complications.

3D-printed distal humeral prosthesis stability relies on periprosthetic soft-tissue reconstruction, particularly of the joint capsule and common tendons of the flexors and extensors, whereas TEA involves the contralateral articular surface with more extensive damage to soft tissues, greater potential for joint instability complications after reconstruction, and prolonged operative time [22]. In addition, in pursuit of better joint stability, our designed distal humeral prosthesis with lateral and medial holes was exactly used to suture the LARS ligament fixation as a whole, which allowed for the more rigid reconstruction of the joint capsule and common tendons of the flexors and extensors over the LARS ligament and through the prosthetic holes. With such delicate soft tissue reconstruction, the stability of the normal elbow joint can be approached. On the contrary, suturing soft tissue directly into smooth holes does not provide enough immediate stability and is prone to suture tearing, and sloughing leading to subluxation during early postoperative exercise [5]. In our case, the LARS ligament wrapping prosthesis as a whole before suturing the soft tissue structures to this whole allows for a more satisfactory immediate fixation, which is conducive to early functional exercise and reduces the risk of early instability. Immediate stabilization of the elbow by suturing the joint capsule, ligaments, and tendons to a hole retained on the prosthesis or to the periprosthetic the LARS ligament is sufficient to allow early flexion and extension, which greatly reduces the risk of joint stiffness [22]. Postoperatively, as the LARS ligament heals progressively with soft tissue, it provides more solid long-term fixation.

## Conclusion

In summary, the 3D-printed modular prosthesis with hemiarthroplasty is a reliable and effective reconstructive strategy. DHH is more acceptable to patients than elbow fusion and TEA in terms of appearance and functional recovery. In cases of large bone defects after wide resection of the neoplastic segment with the presence of an intact ulna to humerus articular surface after pathological fracture of the distal humerus, we believe that DHH based on a 3D-printed personalized healthy side mirror design is the best option to help restore elbow anatomy and function. However, there may still be risks of infection, artificial joint loosening, or nerve injury after surgery, and long-term follow-up is needed. Therefore, to have good outcomes and reduce complications, it is necessary to have a thorough preoperative plan.

## Data Availability

All data generated or analyzed during this study are included in this published article.

## Declarations

### Research Registration

N/A.

## Abbreviations

3D: Three-dimensional
APC: Allograft-prosthetic composite
TEA: Total elbow arthroplasty
DHH: Distal humeral hemiarthroplasty
DICOM: Digital Imaging and Communications in Medicine
LA: Laboratoired’Application et de Recherche Scientifique
MSTS-93: Musculoskeletal Tumor Society- 93
MEP: Mayo Elbow Performance Score
